# Lead, Cadmium, and Arsenic Bioaccessibility of 24 h Duplicate Diet Ingested by Preschool Children Attending Day Care Centers in Brazil

**DOI:** 10.3390/ijerph15081778

**Published:** 2018-08-18

**Authors:** Isabelle Nogueira Leroux, Ana Paula Sacone da Silva Ferreira, Fernanda Pollo Paniz, Tatiana Pedron, Fernanda Junqueira Salles, Fábio Ferreira da Silva, Heloisa França Maltez, Bruno Lemos Batista, Kelly Polido Kaneshiro Olympio

**Affiliations:** 1 Departamento de Saúde Ambiental, Faculdade de Saúde Pública, Universidade de São Paulo, Av. Dr. Arnaldo, 715, Cerqueira César, São Paulo 1246-904, Brazil; isabelle.leroux@usp.br (I.N.L.); saconeap@usp.br (A.P.S.d.S.F.); fjsalles@usp.br (F.J.S.); 2 Centro de Ciências Naturais e Humanas, Universidade Federal do ABC, Avenida dos Estados, 5001, Bairro Santa Terezinha, Santo André—SP—Brasil, Santo André 09210-580, Brazil; fernandapollo@hotmail.com (F.P.P.); tatianapedron@yahoo.com (T.P.); fabiofersil@gmail.com (F.F.d.S.); heloisamaltez@gmail.com (H.F.M.); bruno.lemos@ufabc.edu.br (B.L.B.); 3 Agilent Technologies, Alameda Araguaia, 1142 Alphaville Industrial, Barueri 6455000, Brazil

**Keywords:** bio-accessibility, 24-h diet, preschool children, arsenic intake, cadmium intake, lead intake

## Abstract

Lead, known as a metal with high neurotoxicity to children, cadmium, which is a carcinogenic and bioaccumulative contaminant, and arsenic, a class 1 carcinogenic according to the International Agency for Research on Cancer, are toxic elements (TEs) whose relevant route of exposure may be diet. We determined the bio-accessible fraction of lead, cadmium, and arsenic from the diet of preschool children from two day care centers (DCC). A cross-sectional study was conducted with 64 one–four-year-old children from two DCCs where the 24-h duplicate diet samples were collected. The diet samples were analyzed by ICP-MS for lead, cadmium, and arsenic total concentrations (*n* = 64) and their bio-accessibility were analyzed for a subsample (*n* = 10). The dietary intake (DI) mean for lead, cadmium, and arsenic were 0.18 ± 0.11 µg kg^−1^ bw, 0.08 ± 0.04 µg kg^−1^ bw, and 0.61 ± 0.41 µg kg^−1^ bw, respectively. All DI calculated for TEs, considering total intake, were found lower than the tolerable limits (TL) (European Union, or World Health Organization, WHO, when applicable) except for one child’s Pb intake. Bio-accessibilities ranged between 0% to 93%, 0% to 103%, and 0% to 69%, for lead, cadmium, and arsenic, respectively. Although DI for TEs has been found lower than TL, these reference values have been recently decreased or withdrawn since it was for lead and arsenic whose TL were withdrawn by WHO.

## 1. Introduction

Food is considered an important source of human exposure to some contaminants such as lead [1], cadmium [2,3], and arsenic [4]. Cadmium is one of the 11 metals in the United States Environmental Protection Agency (USEPA) Priority Pollutant List and ingestion of cadmium through contaminated food is the largest source of this metal exposure for non-smokers [3,5,6,7]. Cadmium accumulates and damages the kidneys [2] and is associated with the reduction of childhood cognitive ability [3]. Lead and arsenic, however, are considered the most toxic elements [8] and are also included in the Priority Pollutant List set by USEPA [5]. Lead exposure in childhood is associated with deficits in attention, concentration, intelligence, learning, psychomotor skills, and aggressiveness [9,10,11,12]. Arsenic is also associated with children’s cognitive deficits [13,14]. Food contributes up to 93% of the arsenic total intake [15] and can contribute, on average, toward 83% of lead intake [16].

Children are the most vulnerable group to the effects of potentially toxic elements (PTEs) since they present higher gastrointestinal absorption, faster metabolic processes, detoxification system in development, and higher food consumption by body weight compared to adults. In addition, the blood-brain barrier is not fully developed yet at this stage of life, which allows toxic elements to accumulate in the brain and causes dysfunction in the central nervous system [9,17,18,19].

The concentration of PTEs in foods is not proportional to the bio-accessible concentration [20]. The fraction of a contaminant that is released from the food matrix into the digestive fluid and is, therefore, available for intestinal absorption is the bio-accessible fraction of that component [7]. The bio-accessibility determination of contaminants ingested through food allows a better evaluation of the potential health risks and avoids overestimation [21,22]. Therefore, the aim of this study was to determine, through a 24-h duplicate diet method, the bio-accessible fraction of lead, cadmium, and arsenic from the diet of preschool children attending two day care centers (DCC) in Sao Paulo, Brazil. The Bioaccessible Estimated Daily Intake (BEDI) results were then compared with the Benchmark Dose Level (BMDL) by European Food Safety Authority (ESFSA) for lead and with the Provisional Tolerable Monthly Intake (PTMI) by the World Health Organization (WHO) for cadmium.

## 2. Materials and Methods

### 2.1. Subjects

This study was conducted with 64 one- to four-year-old children from 2 DCCs where one of them is located in the East Zone (coded PF) and the other one is in the South Zone (coded PS) of the city of Sao Paulo, Brazil where high blood lead levels (BLLs) were found in a previous study [23,24]. Children included in this study spend approximately 10 h/day at day care centers. This study was reviewed and approved by the Institutional Review Board (IRB) of the School of Public Health of the University of Sao Paulo, Brazil (Protocol #1.127.698). The children’s parents and guardians were invited to a meeting with the investigators to discuss the potential sources of lead exposure and its health effects. All children whose parents/guardians signed an informed consent form were included in the study.

### 2.2. Sampling of Diets and Preparation

The sampling of diets was conducted with 64 children attending two DCCs. Daily lead, cadmium, and arsenic intake from the diet for each child, considering solid foods and drinks, was analyzed on a weekday using a 24-h duplicate plate method. The parents and guardians were instructed to maintain the usual dietary habits of their children and to duplicate the dietary intake as precisely as possible by observing the amounts that the children really ate and drank. The parents and guardians were asked to use household measures such as a tablespoon, teaspoon, or cupful to approximate the quantities of children’s food ingested. They were also asked to remove the foods’ parts that are not normally eaten such as bones, skin, and seeds before storing the duplicate food and drink in containers in a refrigerator until the researchers collect the 24-h diet samples. For cooked meals, parents were asked to make a similar plate with the same portion of the children’s plates and wait until the children finish the meal and then to add or remove comparable amounts of food from the duplicate plate [25,26]. The same protocol was accomplished at the DCCs and the investigators monitored the children during the whole day. The investigators recorded the portions. The meals served at school were prepared daily and composed of rice, beans, animal protein, vegetables, and fruit. For breakfast, children were served bread or crackers and milk. The school’s menu was elaborated by dieticians. There was a large variability between the foods served at children’s home. After the samples of duplicate diets had been collected, they were transported to the laboratory and thoroughly homogenized using a mixer (Arno model 600 W, Sao Paulo, SP, Brazil). The weight was recorded (Shimadzu, Barueri, SP, Brazil). Diets were aliquoted and stored at −22 °C until the chemical analysis was performed for lead, cadmium, and arsenic.

To avoid contamination, all polypropylene flasks used in the collections were previously cleaned with a detergent solution, rinsed in HNO_3_ 10% overnight, rinsed with deionized water 18.2 MΩ·cm at 25 °C, dried, and stored in a closed polypropylene container. High-purity water produced by a Milli-Q water purification system (Millipore, Bedford, MA, USA) was used throughout. A sub-boiling system (Distilled, Berghof, Germany) was employed to produce high-purity nitric acid.

### 2.3. Sample Preparation

The diet samples were lyophilized by using the lyophilizer (Liotop, L101) at a pressure of 200 μmHg and checked at −50 °C for 48 h. After the lyophilization procedure, the samples were stored at −20 °C.

### 2.4. Acid Digestion for Metals Determination

The elements determination in the diet samples was performed by Inductively Coupled Plasma Mass Spectrometry (ICP-MS). First, 100 mg of lyophilized sample was weighed (in triplicate) and 1 mL of sub-distilled HNO_3_ was added, which was pre-digested during the night (overnight). The pre-digestion was followed by a water bath (Solab SL1522L, Piracicaba, Brazil) at 90 °C at 4 h. After cooling, the volume was made up of 14 mL with deionized water 18.2 MΩ·cm at 25 °C. To verify the accuracy of the procedure, certified reference material (CRM) lobster hepato-pancreas (TORT-3, National Research Council Canada) was used and was prepared by the same procedure.

### 2.5. Bioaccessibility

The samples that presented the highest concentration of PTEs (*n* = 10) were selected to perform the bio-accessibility. In vitro bio-accessibility assessment was performed, according to Bertin et al. [27] and the United States Pharmacopoeia [28]. The assay was performed in two steps: the first step used gastric solution and the second step used intestinal solution. In this phase, the same CRM was used to verify the accuracy of the procedure.

### 2.6. Preparation of Gastrointestinal Solution

For the gastric solution, 0.32 g of pepsin (Sigma-Aldrich, St. Louis, MO, USA) was dissolved in ultra-pure water (~80 mL, Millipore RiO-DITM, Burlington, MA, USA). Afterward, we added 0.7 mL of sub-boiled HCl (36% *v*/*v*, Synth, Diadema, São Paulo, Brazil) and the volume made up to 100 mL. Then, the pH was adjusted to 1.2 using 0.1 M HCl [28].

### 2.7. Preparation of Intestinal Solution

Initially, we solubilized ~0.2 g of bile salts (0.08 g sodium glycodeoxycholate + 0.05 g sodium taurodeoxycholate + 0.08 g sodium taurocholate hydrate) and 0.5 g pancreatin in 100 mL of NaHCO_3_ 3% *w*/*v*. All salts used in the intestinal solution were obtained from sigma Sigma-Aldrich (St. Louis, MO, USA) [28].

### 2.8. Gastric Digestion Simulation

Samples (200 mg) were weighed in conical tubes (50 mL) (Falcon^®^, Corning, Tamaulipas, Mexico). Afterward, we added 3 mL of gastric solution. Then, samples were placed in water bath (SL1522L, Solab, Brazil) at 37 °C during 2 h. The samples were gently shaken every 20 min [27].

### 2.9. Intestinal Digestion Simulation

After gastric digestion, the solution was submitted to the intestinal digestion simulation. For this purpose, NaHCO_3_ (3% *w*/*v*) was added for pH adjustment to 6.8. Then, we added 3 mL of intestinal solution and heated in a water bath at 37 °C for 2 h with shaking (50 rpm). Lastly, the samples were cooled to room temperature and centrifuged (SL700, Solab, Piracicaba, São Paulo, Brazil) at a rate of 1077 G for 20 min. The supernatants of this step were separated from the precipitates. Precipitates and supernatant were digested following the same procedure previously described for totals element quantification [27].

### 2.10. Metals Determination

The determination of the elements total concentration and their bio-accessibilities in diet samples were carried out by inductively coupled plasma mass spectrometry (ICP-MS) (Agilent Technologies, 7900, Hachioji, Japan). An external calibration curve was prepared with standard multi-element solution (PerkinElmer, Inc., Waltham, MA, USA) at concentrations of 0.1 μg L^−1^, 1 μg L^−1^, 5 μg L^−1^, 10 μg L^−1^, 50 μg L^−1^, 100 μg L^−1^, 200 μg L^−1^, 500 μg L^−1^, and 1000 μg L^−1^. Blank solutions were also prepared and lobster hepato-pancreas reference material (TORT-3, National Research Council Canada) was prepared using the same protocol for samples. The ICP-MS conditions are presented in Table 1. The limits of detection were 0.003 μg L^−1^, 0.001 μg L^−1^, and 0.006 μg L^−1^ for Pb, Cd, and As, respectively. The recovery for the reference material were 89%, 94%, and 120% for Pb, Cd, and As, respectively.

## 3. Results

The anthropometric characteristics of children are presented in Table 2.

The bio-accessibility fractions of lead, cadmium, and arsenic are presented in Table 3. Even though some samples have presented the bio-accessible percentage close to 100%, none of the samples reached the values of BMDL, PTMI, or the withdrawn PTWI of the PTEs studied. 

Lead and cadmium daily intake were similar in both DCCs (Table 4). The mean lead intake values were below the European Union BMDL for the development of neurotoxic effects in children, which corresponds to 36% of BMDL for both genders. A two-year-old boy attending the DCC PS ingested a concentration 37.5% (0.8 μg Pb kg^−1^ bw) higher than the BMDL (0.5 μg Pb kg^−1^ bw). His diet was one of the samples assessed for bio-accessible fractions. Lead was 8.19% bio-accessible in his diet, which corresponds to an intake of 0.07 μg kg^−1^ bw. Therefore, the total lead intake of this boy was higher than the BMDL, but the bio-accessible fraction was not and it reached about 13% of the TI. 

## 4. Discussion

As far as we know, the present study was the first one to determine the bio-accessibility of metals in a 24-h total duplicate diet. Previous studies evaluated only the elemental bio-accessibility in food, which was raw and/or cooked, and did not consider the total diet. 

According to the Joint Expert Committee on Food Additives (JECFA), the provisional tolerable weekly intake (PTWI) for inorganic arsenic (15 μg kg^−1^ bw per week or 2.1 μg kg^−1^ bw per day) was withdrawn [29] as well as PTWI for lead (25 μg kg^−1^ bw) [30]. The provisional tolerable monthly intake (PTMI) for cadmium is 25 μg kg^−1^ bw per month [31]. The withdrawing of the tolerable intake values for lead and arsenic in 2011 means that the tolerable values once established by JECFA were not considered secure anymore. Therefore, it is not possible to establish a new tolerable value that would be considered safe. The European Union has the Benchmark Dose Level (BMDL) for lead, which is 0.5 μg Pb kg^−1^ bw [32]. However, for cadmium [33] and arsenic [34], the European Union values were also withdrawn. 

The highest Bio-accessible Estimated Daily Intake (BEDI) corresponded to 34% of Pb BMDL [32] and 9.2% of Cd PTMI [31] while, for As, BEDI is equivalent to 59% of the withdrawn PTWI [31]. The wide range in bio-accessible fractions (Table 3) are explained with the variability in children’s diet. Each child consumed an exclusive arrangement of specific food and portions, which leads to different PTEs concentrations and also different bio-accessible concentrations.

Hu et al. [35] determined lead and cadmium bio-accessibility in vegetables cultivated in Hong Kong and they found a range of bio-accessibility from 20% to 68% and from 21% to 96%, respectively. Fu and Cui [20] also verified the bio-accessibility for lead and cadmium in vegetables and evaluated the differences in the gastric and intestinal phases from raw and cooked food. They found that cadmium is more bioavailable in the gastric phase. Lead, in the intestinal phase, and cooked vegetables presented lower concentrations of these bio-accessible elements. For lead, the bio-accessibility mean was 9.4% in raw vegetables and 3.2% in cooked vegetables while cadmium was 11.2% in raw vegetables and 6.1% in cooked vegetables.

Regarding daily total intake, values higher than those found in the present study were reported by Kim et al. [36] whom investigated the exposure to lead and cadmium of 457 South Korean children aged 0–6 years old through a two-day 24-h recall, which is a different method. The mean of the lead intake was 0.46 μg kg ^−1^ bw per day with 35% of children exceeding the BMDL value of 0.5 μg Pb kg^−1^ bw per day. Fruits and milk appeared as the main sources of lead exposure. For cadmium, the mean intake was 0.34 μg Cd kg^−1^ bw per day. Cereals, fish, shellfish, and algae had a significant contribution for the intake of cadmium. 

Watanabe et al. [37] reported that the exposure to lead and cadmium through the 24-h duplicate diet and urine evaluation of 108 children (4–6 years old) from 4 DCC located in Seoul city and Jeju Island, Korea. They found a geometric mean for Cd intake of 0.58 μg kg^−1^ bw per day, which is seven times higher than our findings (Table 4) and 19.5% of this was attributed to rice consumption. For Pb, a geometric mean of 0.27 μg Pb kg^−1^ bw per day was found, which is 1.5 times higher than our findings (Table 4). 

Pysz et al. [38,39] evaluated lead and cadmium intake of four- to six-year-old children and adolescents who lived in orphanages in Krakow, Poland through a 24-h duplicate diet of four days in each season of the year including weekend days. For the children from the orphanage which has an age range equivalent with the children in the present study, lead and cadmium annual mean intake were 11.57 μg kg^−1^ bw per week and 16.63 μg kg^−1^ bw per week, respectively. These values are higher than the ones found in the present study and correspond to concentrations 69.75% above BMDL for Pb [32] and 62.42% above the PTMI for Cd [31]. 

A duplicate diet approach was also applied in a study performed in Jinhu area, China to estimate the arsenic dietary intake for 30 children (two- to five-years-old) and 30 adults (29–55 years old). The diet collection period were three days wherein one of them was a weekend day. The mean arsenic intake for the children was similar to our findings (Table 4), 0.6 mg kg^−1^ bw day^−1^ [1].

Concerning arsenic food sources such as rice and seafood, the main source for this study population would be rice since Brazilian seafood consumption is quite low when compared with other countries [40] and it was almost not seen in the analyzed children’s diets. Rice consumption was identified as a source of arsenic exposure for children in the United States of America [41]. Besides that, in Brazil, rice is widely consumed and it is a food with great importance in the country’s feeding habit. Batista et al. [42] determined arsenic species’ concentrations in different types of rice produced in Brazil. The mean concentration they found in white rice, the type which was consumed by children in the present study, was 223 ng g^−1^. This arsenic concentration was composed of 50% of inorganic arsenic, the most toxic one.

For children, chemicals exposure is a huge threat due to the children’s health impact on organs, systems, and functions because of their developmental process and growth [43]. Since the presence of PTEs in children diets is related to the food contamination through soil or air pollution, the difference between the PTEs concentration in this study and the others cited can be attributed to different environmental contamination levels around the world. The soil contamination is related to some activities as mining, industrial, or even agriculture activities. Then, the plants and food that grows in contaminated soil absorb and accumulate the PTEs [20,35]. Additionally, the atmospheric particulate material that sets down on plants’ surfaces can contaminate them. Xiong et al. [22] found that vegetables are high in lead and cadmium concentrations originating from air pollution. Industrial particulate contributes to 25% to 40% of total PTEs concentration, which deposits on plants’ leaves and is absorbed through their vascular system. Waste incineration and traffic flow contribute to particulate material emission as well [22]. The diet is considered a relevant source for some toxicants. However, it is not the unique one and it requires a global approach to control the chemical exposure. Children are exposed to many toxic materials at home, at school, on the playground, and other places [24,43,44,45,46,47]. The maximum limits for toxic materials are continuously decreasing due to regulation agencies and the implementation of specific regulations for children’s items [48]. The Canada Consumer Product Safety Act (CCPS) regulates children’s jewelry items, which contain lead and cadmium [49]. Furthermore, the children’s cosmetics items such as fragrances, makeup, nail polish, face paint, and similar items are regulated by Health Canada under the Cosmetic Regulations of the Food and Drugs Act [50]. Similar regulations of the lead concentration in paint or surface coating on children’s toys have been established in the European Union and Australia [10,51].

The present study brings important data related to children’s exposure to highly PTEs as lead, cadmium, and arsenic through diet. However, some limitation might be considered. We evaluated one weekday. Future studies may include more days to consider the variety of the meals consumed by children. Taking into account the weekend, the diet can be different once the children are not attending day care centers.

## 5. Conclusions

Our findings showed that Brazilian preschool children’s diet did not contain high arsenic, cadmium, and lead levels compared to data from other countries. Even though our findings indicate that children’s dietary exposure to arsenic, cadmium, and lead is not very high, the bio-accessibility range of the elements had a large variability and the safe reference limits have been decreased or withdrawn. Considering a possible overall exposure, with other further exposure sources and routes, our findings suggest that the children may be at considerable risk of lead and arsenic exposure through diet. Currently, especially for arsenic, neither WHO nor EFSA has benchmarks considered safe for its ingestion. We believe the same should be proposed for lead. There is no safe level for lead exposure. 

## Figures and Tables

**Table 1:** Operational conditions for ICP-MS in total diets analyses and bio-accessibility.

Parameter	Diet	Bio-accessibility
Radio Frequency Power	1600 W	1550 W
Argon Flow Rate	15 L min^−1^	15 L min^−1^
He Flow	5.0 mL min^−1^	5.0 mL min^−1^
HeHE	10 mL min^−1^	10 mL min^−1^
Nebulizer Gas Flow Rate	0.68 L min^−1^	1.05 L min^−1^
Collision Cell	Helium (purity > 99.99%)	Helium (purity > 99.99%)
Nebulizer Chamber	Scott (double pass)	Scott (double pass)
Interface	Nickel cones	Nickel cones
Sampling Cone	1 mm	1 mm
Skimmer	0.45 mm	0.45 mm

**Table 2:** Anthropometric characteristics of children from the day care centers (DCC) PS and PF (São Paulo, 2015).

	PS DCC		PF DCC		Total	
	Male	Female	Male	Female	Male	Female
	(*n* = 26)	(*n* = 15)	(*n* = 8)	(*n* = 15)	(*n* = 34)	(*n* = 30)
	Mean ± SD	Mean ± SD	Mean ± SD	Mean ± SD	Mean ± SD	Mean ± SD
Age (years)	3.6 ± 0.6	3.3 ± 0.7	2.6 ± 0.9	3.4 ± 0.7	3.4 ± 0.8	3.4 ± 0.7
Height (cm)	98 ± 7	95 ± 6	93 ± 12	101 ± 6	97 ± 8	98 ± 7
Weight (kg)	17 ± 4	15 ± 2	15 ± 4	17 ± 3	16 ± 4	16 ± 3
BMI * (kg/m^2^)	17 ± 2	17 ± 1	18 ± 1	16 ± 2	17 ± 2	17 ± 2

* Body mass index.

**Table 3:** Percentage range of bio-accessibility (minimum–maximum) for lead, cadmium, and arsenic in diet (*n* = 10). Range of Children’s Bio-accessible Estimated Daily Intake (BEDI) per body weight (μg kg^−1^ bw^−1^ day) and BEDI Quartiles Q1, Q2, Q3, and Q4 (μg kg^−1^ bw per day) São Paulo, 2015.

Parameter	Lead	Cadmium	Arsenic
Range of Bio-accessibility (%)	0–93	0–100	0–69
Range of BEDI (μg kg^−1^ bw per day)	0–0.17	0–0.04	0–1.26
BEDI Median (μg kg^−1^ bw per day)	0.07	0.03	0.10
BEDI Q1 (μg kg^−1^ bw per day)	0.02	0.02	0.08
BEDI Q2 (μg kg^−1^ bw per day)	0.07	0.03	0.10
BEDI Q3 (μg kg^−1^ bw per day)	0.09	0.03	0.27
BEDI Q4 (μg kg^−1^ bw per day)	0.17	0.04	1.26

**Table 4:** Children’s total daily intake of lead (Pb), arsenic (As), and cadmium (Cd) (μg kg^−1^ bw^−1^ day) from two day care centers of São Paulo (PS DCC and PF DCC), 2015.

	PS DCC		PF DCC		Total	
	Male	Female	Male	Female	Male	Female
	(*n* = 26)	(*n* = 15)	(*n* = 8)	(*n* = 15)	(*n* = 34)	(*n* = 30)
	Mean ± SD	Mean ± SD	Mean ± SD	Mean ± SD	Mean ± SD	Mean ± SD
Pb daily intake	0.18 ± 0.15	0.20 ± 0.08	0.18 ± 0.08	0.15 ± 0.05	0.18 ± 0.14	0.18 ± 0.07
As daily intake	0.70 ± 0.35	0.79 ± 0.49	0.54 ± 0.55	0.35 ± 0.19	0.66 ± 0.40	0.57 ± 0.43
Cd daily intake	0.08 ± 0.03	0.10 ± 0.05	0.08 ± 0.03	0.07 ± 0.03	0.08 ± 0.03	0.09 ± 0.04
